# Supplementary material for: Modeling glioblastoma heterogeneity as a dynamic network of cell states
Source: Mol Syst Biol. 2021 Sep 16;17(9):e10105. doi: 10.15252/msb.202010105 (PMC8444284; doi:10.15252/msb.202010105)
Supplement: Supplementary file 6 — Source Data for Figure 5 [file MSB-17-e10105-s004.zip › Figure5A_sourcedata/GSEA_3071/hallmarks_stateVI.GseaPreranked.1623742557031/HALLMARK_COAGULATION.html]

Details for gene set HALLMARK\_COAGULATION[GSEA]

|  || Dataset | state63071 |
| Phenotype | NoPhenotypeAvailable |
| Upregulated in class | na\_neg |
| GeneSet | HALLMARK\_COAGULATION |
| Enrichment Score (ES) | -0.27847937 |
| Normalized Enrichment Score (NES) | -1.1343563 |
| Nominal p-value | 0.3178392 |
| FDR q-value | 0.5591313 |
| FWER p-Value | 1.0 |
Table: GSEA Results Summary

  

Fig 1: Enrichment plot: HALLMARK\_COAGULATION      
 Profile of the Running ES Score & Positions of GeneSet Members on the Rank Ordered List

  

| PROBE | GENE SYMBOL | GENE\_TITLE | RANK IN GENE LIST | RANK METRIC SCORE | RUNNING ES | CORE ENRICHMENT || 1 | SERPINE1 |  |  | 0 | 1.673 | 0.0690 | Yes |
| 2 | THBS1 |  |  | 12 | 1.097 | 0.1112 | Yes |
| 3 | F3 |  |  | 26 | 0.988 | 0.1483 | Yes |
| 4 | CAPN2 |  |  | 67 | 0.818 | 0.1710 | Yes |
| 5 | ITGA2 |  |  | 71 | 0.810 | 0.2036 | Yes |
| 6 | PRSS23 |  |  | 108 | 0.732 | 0.2237 | Yes |
| 7 | DUSP14 |  |  | 111 | 0.721 | 0.2529 | Yes |
| 8 | MAFF |  |  | 253 | 0.562 | 0.2370 | No |
| 9 | FBN1 |  |  | 311 | 0.536 | 0.2432 | No |
| 10 | CTSB |  |  | 471 | 0.464 | 0.2182 | No |
| 11 | WDR1 |  |  | 540 | 0.444 | 0.2177 | No |
| 12 | PEF1 |  |  | 547 | 0.442 | 0.2342 | No |
| 13 | CSRP1 |  |  | 671 | 0.407 | 0.2169 | No |
| 14 | TFPI2 |  |  | 727 | 0.391 | 0.2177 | No |
| 15 | GNG12 |  |  | 1178 | 0.319 | 0.1060 | No |
| 16 | TIMP3 |  |  | 1290 | 0.305 | 0.0878 | No |
| 17 | FN1 |  |  | 1654 | 0.264 | -0.0021 | No |
| 18 | FURIN |  |  | 1702 | 0.260 | -0.0044 | No |
| 19 | CTSO |  |  | 2060 | -0.288 | -0.0916 | No |
| 20 | FYN |  |  | 2118 | -0.301 | -0.0950 | No |
| 21 | C1R |  |  | 2388 | -0.351 | -0.1552 | No |
| 22 | MMP14 |  |  | 2440 | -0.365 | -0.1543 | No |
| 23 | MMP2 |  |  | 2550 | -0.388 | -0.1686 | No |
| 24 | PROS1 |  |  | 2647 | -0.411 | -0.1783 | No |
| 25 | P2RY1 |  |  | 2772 | -0.447 | -0.1942 | No |
| 26 | CAPN5 |  |  | 2809 | -0.460 | -0.1852 | No |
| 27 | GSN |  |  | 3041 | -0.559 | -0.2263 | No |
| 28 | CRIP2 |  |  | 3230 | -0.697 | -0.2497 | No |
| 29 | HTRA1 |  |  | 3331 | -0.796 | -0.2446 | No |
| 30 | SPARC |  |  | 3356 | -0.822 | -0.2174 | No |
| 31 | PLAT |  |  | 3416 | -0.927 | -0.1955 | No |
| 32 | CD9 |  |  | 3445 | -0.995 | -0.1622 | No |
| 33 | A2M |  |  | 3503 | -1.122 | -0.1318 | No |
| 34 | PDGFB |  |  | 3506 | -1.131 | -0.0857 | No |
| 35 | DUSP6 |  |  | 3512 | -1.151 | -0.0396 | No |
| 36 | CLU |  |  | 3601 | -1.801 | 0.0103 | No |
Table: GSEA details [plain text format]

  

Fig 2: HALLMARK\_COAGULATION: Random ES distribution      
 Gene set null distribution of ES for **HALLMARK\_COAGULATION**
